# *Lagenidium giganteum* Pathogenicity in Mammals

**DOI:** 10.3201/eid2102.141091

**Published:** 2015-02

**Authors:** Raquel Vilela, John W. Taylor, Edward D. Walker, Leonel Mendoza

**Affiliations:** Michigan State University, East Lansing, Michigan, USA (R. Vilela, E.D. Walker, L. Mendoza);; Universidade Federal do Minas Gerais, Belo Horizonte, Brazil (R. Vilela);; University of California, Berkeley, California, USA (J.W. Taylor)

**Keywords:** Oomycetes, Oomycota, fungal-like, Lagenidium, Lagenidium giganteum, Pythium insidiosum, pythiosis, lagenidiosis, biological control, fungi

## Abstract

Infections of mammals by species in the phylum Oomycota taxonomically and molecularly similar to known *Lagenidium giganteum* strains have increased. During 2013–2014, we conducted a phylogenetic study of 21 mammalian *Lagenidium* isolates; we found that 11 cannot be differentiated from *L. giganteum* strains that the US Environmental Protection Agency approved for biological control of mosquitoes; these strains were later unregistered and are no longer available. *L. giganteum* strains pathogenic to mammals formed a strongly supported clade with the biological control isolates, and both types experimentally infected mosquito larvae. However, the strains from mammals grew well at 25°C and 37°C, whereas the biological control strains developed normally at 25°C but poorly at higher temperatures. The emergence of heat-tolerant strains of *L. giganteum* pathogenic to lower animals and humans is of environmental and public health concern.

## Abstract 2

Strains pathogenic to mammals share phylogenetic and phenotypic features with strains approved for mosquito control.

During the 20th century, *Pythium insidiosum* was the only fungus-like species from the phylum Oomycota known to cause life-threatening infections in mammals and birds (*1*,*2*). In 2003, a novel group of pathogenic Oomycota was isolated in the southeastern United States from dogs with invasive cutaneous infections resembling pythiosis, but the strains had morphologic and molecular affinities with the genus *Lagenidium* (*3*,*4*). Subsequently, other strains similar to previously studied *Lagenidium* species were found to be associated with infections in dogs, cats, and humans (*5*–*7*). The disease caused by these 2 Oomycota species has been reported mainly in apparently healthy hosts; thus, these species’ evolutionary traits need to be investigated (*1*–*7*). Although detailed descriptions of the clinical, pathologic, and serologic features of *Lagenidium* spp. pathogenic to mammals have been published (*3*,*4*), their taxonomic relationship to *Lagenidium* strains approved for biological control and the capacity of their zoospores to infect mosquitoes have yet to be investigated (*5*,*6*).

The genus *Lagenidium*, introduced by Schenk in 1857 (*8*), comprises numerous saprotrophic species (*9*), as well as several members that are pathogenic to algae, phytoplankton, pollen, barnacles, blue crabs, mosquito larvae, shrimp, and mammals (*3*–*5*). Couch (*10*) isolated *L. giganteum* from *Daphnia* larvae in Virginia, USA. Later, Couch and Romney (*11*) confirmed that *L. giganteum* infects and kills mosquito larvae as it reproduces sexually. Their work stimulated interest in *L. giganteum* as a biological control agent of mosquitoes (*11*,*12*). In 1995, the US Environmental Protection Agency registered *L. giganteum* under the trade name Laginex as a biocontrol agent (*13*) but later deregistered it at the request of the manufacturer (http://www.gpo.gov/fdsys/pkg/FR-2011-09-28/pdf/2011-24832.pdf). *L. giganteum* strains used for testing and commercial development were deposited within the American Type Culture Collection (ATCC), and the ATCC strains and other not-well-characterized strains were released in nature for field studies (*12*–*16*). In this study, conducted during 2013–2014, we investigated 21 mammalian *Lagenidium* strains and compared them with *L. giganteum* isolates used for mosquito control.

## Materials and Methods

### Living Cultures

A complete list of the strains and their accession numbers is shown in the Table. In brief, we studied the following isolates: *L. giganteum* (ATCC 36492 = ATCC 52675 used as biological control and ATCC 48336 isolated from mosquito larvae); *L. humanum* (ATCC 76726 isolated from the skin of dead humans); *L. giganteum* pathogenic to mammals (culture collection at the Biomedical Laboratory Diagnostics, Michigan State University (MTLA): MTLA 01 = ATCCMYA-4933 type strain, MTLA-03, MTLA-04 = ATCCMYA-4934 (from a US cat), MTLA-05 = ATCCMYA-4935, MTLA-10, MTLA-12, MTLA-14, MTLA-15, MTLA-16, MTLA-17, and MTLA-18 from dogs); *L. ajelloi* (MTLA-06 = ATCCMYA-4936 type strain, MTLA-07 = ATCCMYA-4937, MTLA-19, MTLA-20, MTLA-21, MTLA-22, MTLA-23, isolated from dogs); *L. vilelae* (MTLA-24 = DNA extracted from fixed feline tissue and MTLA-25 cat strain); and *L. albertoi* (MTLA13 = ATCCMYA-4932 type strain).

**Table:** Strains, accession numbers, acronyms, optimal temperatures, and affected hosts on the studied *Lagenidium* isolates studied, 2011–2012

Taxonomic name	Strain identification†	Acronym	Primer					Optimal temperature, °C	Host
			Internal transcriber spacer	CDC42	COXII	HSP90	TUB		
*L. giganteum*	NPI01	Lgm1	EF016915	0	0	0	0	25	Mosquito larvae
*L. giganteum*	ATCC 48336	Lgm2	JQ745259	0	0	0	0	25	Mosquito larvae
*L. giganteum*	ATCC 36492	Lgm3	AY151183	JX985758	AF086697	JX999093	JX999124	25	Mosquito larvae
*Lagenidium* sp.	CBS 127533	Ls1	HQ395647	0	0	0	0	ND	Nematodes
*Lagenidium* sp. A	CBS 126881	Lspa1	HQ111460	0	0	0	0	ND	Nematodes
*Lagenidium* sp .A	CBS 126885	Lspa2	HQ111452	0	0	0	0	ND	Nematodes
*Lagenidium* sp .A	CBS 126878	Lspa3	HQ111464	0	0	0	0	ND	Nematodes
*Lagenidium* sp. A	CBS 126880	Lspa4	HQ111459	0	0	0	0	ND	Nematodes
*Lagenidium* sp .B	CBS 126882	Lspb1	HQ111461	0	0	0	0	ND	Nematodes
*Lagenidium* sp. B	CBS 126879	Lspb2	HQ111463	0	0	0	0	ND	Nematodes
*Lagenidium* sp. B	CBS 126884	Lspb3	HQ111450	0	0	0	0	ND	Nematodes
*Lagenidium* sp. B	CBS 126883	Lspb4	HQ111462	0	0	0	0	ND	Nematodes
*L. callinectes*	NJM-0531	Lc1	AB285488	0	0	0	0	25	Crustacea
*L. callinectes*	NJM-8989	Lc2	AB285487	0	0	0	0	25	Crustacea
*L. callinectes*	ATCC 24973	Lc3	AB285486	0	0	0	0	25	Crustacea
*L. vilelae*	CBS 127042	Lv1	HQ111455	0	0	0	0	ND	Nematodes
*Lagenidium* sp. C	CBS 127284	Lspc1	HQ111472	0	0	0	0	ND	Nematodes
*Lagenidium* sp. C	CBS 127285	Lspc2	HQ111470	0	0	0	0	ND	Nematodes
*Lagenidium* sp. C	CBS 127283	Lscd1	HQ111471	0	0	0	0	ND	Nematodes
*Lagenidium* sp. C	CBS 127276	Lspc3	HQ111451	0	0	0	0	ND	Nematodes
*Lagenidium* sp. C	CBS 127281	Lspd2	HQ111473	0	0	0	0	ND	Nematodes
*Lagenidium* sp. D	CBS 127277	Lspd3	HQ111454	0	0	0	0	ND	Nematodes
*L. caudatum*	CBS 58485	Lca1	HQ643136	0	AF290309	0	0	25	Nematodes
*L. myophilum*	NJM-8403	Lm1	AB285497	0	AF290311	0	0	20	Shrimp
*L. humanum*	ATCC 76726	Lh1	JX970867	JX985757	AF290310	JX999094	JX999125	25	Saprotrophic
*L. giganteum mammals*	ATCCMYA-4933	Lgp1	JF919611	JF919613	JX970853	JX999097	JX999127	37°C fast	Dog
*L. giganteum mammals*	MTLA-03	Lgp2	JX999099	JF919612	JX970852	JX999099	JX999128	37 fast	Dog
*L. giganteum mammals*	ATCCMYA-4934	Lgp3	JX999096	JF919613	JX970854	JX999096	JX999126	37 slow	Cat
*L. giganteum mammals*	ATCCMYA-4935	Lgp4	JX999098	JF919614	JX970855	JX999098	JX999129	37 fast	Dog
*L. giganteum mammals*	MTLA-10	Lgp5	KJ506111	KJ506076	KJ506083	KJ506097	KJ506111	37 fast	Dog
*L. giganteum mammals*	MTLA-12	Lgp6	KJ506112	KJ506078	KJ506084	KJ506098	KJ506112	37 fast	Dog
*L. giganteum mammals*	MTLA-14	Lgp7	KJ506113	KJ506077	KJ506085	KJ506099	KJ506113	37 fast	Dog
*L. giganteum mammals*	MTLA-15	Lgp8	KJ506116	KJ506081	KJ506087	KJ506103	KJ506116	37 fast	Dog
*L. giganteum mammals*	MTLA-16	Lgp9	KJ506115	KJ506080	KJ506086	KJ506102	KJ506115	37 fast	Dog
*L. giganteum mammals*	MTLA-17	Lgp10	KJ506117	KJ506082	KJ506088	KJ506101	KJ506117	37 fast	Dog
*L. giganteum mammals*	MTLA-18	Lgp11	KJ506114	JX970841	KJ506089	KJ506100	KJ506114	37 fast	Dog
*L. ajelloi*	ATCCMYA-4936	La1	JX970885	JX970885	JX970841	JX999112	JX999143	37 slow	Dog
*L. ajelloi*	ATCCMYA-4937	La2	JX970884	JX970884	JX970842	JX999113	JX999144	37 slow	Dog
*L. ajelloi*	MTLA-19	La3	KJ506134	KJ506070	KJ506092	KJ506106	KJ506120	37 slow	Dog
*L. ajelloi*	MTLA-20	La4	KJ506135	KJ506071	KJ506094	KJ506109	KJ506124	37 slow	Dog
*L. ajelloi*	MTLA-21	La5	KJ506137	KJ506073	KJ506095	KJ506108	KJ506122	37 slow	Dog
*L. ajelloi*	MTLA-22	La6	KJ506138	KJ506069	KJ506096	KJ506107	KJ506123	37 slow	Dog
*L. ajelloi*	MTLA-23	La7	KJ506136	KJ506072	KJ506093	KJ506110	KJ506121	37 slow	Dog
*L. albertoi*	ATCCMYA-4932	Lal8	JX970870	JX970838	JX970838	JX999115	JX999142	37 slow	Human
*L. vilelae*	MTLA-24	Lv2	KJ506133	KJ506075	KJ506091	KJ506105	KJ506119	37 slow	Cat
*L. vilelae*	MTLA-25-Tissue	Lv3	KJ506132	KJ506090	KJ506090	KJ506104	KJ506118	ND	Cat
*Pythium insidiosum*	MTPI-36	Pi1	AY486144	0	0	0	0	37	Mammals
*P. grandisporangium*	ATCC 28295	Pg1_c	AY151182	0	0	0	0	25	Saprotrophic
*P. aphanidermatum*	STR-135	Pa1_a	AY151180	0	0	0	0	25	Plants
*P. diliense*	STR-66	Pd1_a	AY151181	0	0	0	0	25	Plants
*P. flevoense*	STR-W041	Pf1_b	EU240168	0	0	0	0	25	Plants/fish
*P. diclinum*	STR-P7824	Pdi1_b	EF153675	0	0	0	0	25	Plants
*P. capillosum*	CBS 222–94	Pc1_b	AY598635	0	0	0	0	25	Saprotrophic
*P. sylvaticum*	CBS 453.67	Ps1_f	AY598657	0	0	0	0	25	Plants
*P. pleroticum*	CBS 776.81	Pp1_e	AY598642	0	0	0	0	25	Plants
*P. iwayamai*	CBS 156.64	Piw1_g	AY598648	0	0	0	0	25	Plants
*P. undulatum*	CBS 157.69	Pu1_j	AY598708	0	0	0	0	25	Plants
*P. ultimum*	CBS 398.51	Pul1_i	AY598642	0	0	0	0	25	Plants
*P. nunn*	CBS 808.96	Pn1_j	AY598709	0	0	0	0	25	Fungi

*ATCC, American Type Culture Collection; CBS, Centraalbureau voor Schimmelcultures; CDC42, cell division cycle 42; COXII, cytochrome oxidase II; HSP90, heat shock protein 90; ND, no data; TUB, tubulin; 0, no DNA sequences available. †MTLA and MTPI strains Biomedical Laboratory Diagnostics, Michigan State University culture collection.

### Media and Culture Conditions

The isolates were grown on brain-heart infusion (BHI) (DIFCO, Detroit, MI, USA), corn meal agar (CMA) (BBL, Sparks, MD, USA), 2% Sabouraud dextrose agar (SDA), 2% Sabouraud dextrose broth, in triplicate. Cardinal temperatures of growth (25°C, 30°C, and 37°C measured during 3 days’ incubation) and their relation to the production of sexual and asexual structures were evaluated on the above media. Development of the different stages of vesicle and zoospore formation, cleavage, and release were assessed on colonized grass leaves in water cultures containing Ca^++^ and Mg^++^. Briefly, the evaluated strains (Table) were subcultured on SDA plates at 37°C for 24 h. After incubation, 5 × 5–mm diameter blocks were cut from the advancing edges of the culture and placed on top of a 2% water agar plates. Sterile 4 × 10–mm grass blades were laid on top of each block and incubated at 37°C for 24 h (*L. giganteum* = MTLA-01 from mammals). The grass blades were then collected and placed in a beaker that contained 50 mL of sporulation solution (made of 2 solutions: mix no. 1 contained NH_42_HPO_4_ [66.04 g], KH_2_PO_4_ [68.05 g], and K_2_HPO_4_ [87.09 g] in 500 mL H_2_O; and mix no. 2 contained CaCl_2_.2H_2_O [18.38 g] and MgCl_2_.6H_2_O [25.42 g] in 250 mL H_2_O). The sporulation solution was obtained by mixing 0.5 mL of solution no. 1 plus 0.1 mL of solution no. 2 in 1.0 L of distilled water. The beaker with the 50-mL of sporulation solution plus the parasitized grass blades was incubated at 37°C, and the development of sporangia and zoospores microscopically was evaluated every 30 min for the following 6 h (or longer when needed).

### Morphologic Description of *L. giganteum* from Mammals

We evaluated the morphologic features of *L. giganteum* strains after subculture on BHI, CMA, and SDA at different intervals during 5 days’ incubation at 37°C or at room temperature (25°C). Briefly, 4 × 4–mm agar blocks were cut from the above media and mixed with 1 drop of lactophenol cotton blue (phenol 20 mL, lactic acid 20 mL, glycerol 40 mL, and distilled water 20 mL). The morphologic features of their hyphal and other structures developed in these media, including oogonia, were microscopically investigated. We evaluated the development of sporangia and zoospores in sporulation medium (see above). After incubation at the induction temperatures, the beaker containing 50 mL of sporulation medium plus the parasitized grass blades was inspected on an inverted microscope. If vesicles and zoospores developed, we removedthe grass blade with >10 vesicles, using tweezers, and placed it on a glass slide. Immediately, 5 μL of Merthiolate (0.02%) was added to the grass blade to stop the movement of the zoospores and facilitate measurements. Alternatively, grass blades containing vesicles with zoospores were also transferred to plates holding *Culex pipiens* larvae.

### Experimental Mosquito Infection

The strains of *L. giganteum* from mammals (MTLA01, MTLA03, MTLA04, MTLA05, and MTLA10) (Table) were used to evaluate their capability to infect mosquito larvae of *C. pipiens*. Instar 3 *C. pipiens* larvae were collected and placed in 6-well plates (1 plate per experiment; Corning Inc., Corning, NY, USA) containing 5 mL of water and fed every other day with fish food flakes (TetraMin, Lancaster, PA, USA) until the end of the experiment. We transferred fresh grass blades, with >10 vesicles per blade containing numerous zoospores, to the plates holding 5 mosquito larvae per well (total of 30 larvae per plate). The plates were incubated at 25°C and observed daily by using an inverted microscope for the next 3 weeks. The criteria to select putative infected mosquito larvae were based on their swimming capabilities. We closely inspected slower swimmers for filamentous structures outside and between the larval segments and inside their bodies. Every trial included its positive (*L. giganteum* biological control ATCC 36492 = ATCC 52675) and negative controls (larvae without exposure to *L. giganteum* species), and the experiment was repeated twice (total 60 *C. pipens* larvae).

### DNA Extraction and Molecular Procedures

The 20 strains investigated (Table) were inoculated into 250-mL flasks containing 100 mL of Sabouraud dextrose broth and incubated for 72 h at 37°C on a shaker rotating at 150 rpm. In addition, 1 formalin-fixed tissue sample from a cat was also used to extract total genomic DNA following the company protocols (QIAGEN, Valencia, CA, USA). After incubation, the cultures were killed with Merthiolate (0.02%, wt/vol) and then filtrated to separate the hyphal cell mass. The hyphal cell mass was then transferred to a mortar and ground in the presence of liquid nitrogen. DNA from the disrupted hyphae was treated with sodium dodecyl sulfate and proteinase K and then incubated at 60°C for 1 h, and genomic DNA of the hydrae was extracted with phenol, chloroform, isoamyl alcohol (Sigma, St. Louis, MO. USA). PCR was conducted by hot start amplification using the following primers: the universal primers for internal transcribed spacers (ITS): ITS1 = TCCGTAGGTGAACCTGCGG and ITS4 = TCCTCCGCTTATTGATATGC; cytochrome oxidase II (COXII): COX.LagF = 5′-CCACAAATTTCACTACATTGA-3′ and COX.LagR = 5′-TAGGATTTCAAGATCCTGC-3′; heat shock protein 90 (HSP90): HSP90.LagF = 5′-CAACCTBGGHACSATYGCCAAG-3′ and HSP90.LagR = 5′-ACRAAMGACARGTAYTCVGGCA-3′; cell division cycle 42 (CDC42): CDC42.LagF = 5′-GTSCCVACYGTVTTYGANAAYTA-3′ and CDC42.LagR = 5′-GCWSWGCAYTCVASRTAYTT-3′; Tubulin: TUB.LagF = 5′-GGTGGTGGTACCGGTTC-3′ and TUB.LagR = 5′-GACACACGCTTGAACATC-3′. In addition, the following primers were constructed to PCR amplify some of the *L. ajelloi* and *L. vilelae* strains: CDC42.Lag2F = 5′-GTGCCGACYGTGTTYGABAAC-3′ and CDC42.Lag2R = 5′-CTSTTCKGTTGTRATBGG-3′; and HSP90.Lag2F = 5′-GAGGCCTTCGTGGAAGCG-3′ and HSP90.Lag2R = 5′-GTTGTTCATTTTCTTGCGGG-3′. The PCR temperature-cycling parameters were as follows: 10 min at 95°C and 1 min for subsequent cycles, annealing for 1 min at 60°C, and elongation at 72°C for 2 min. The parameter was repeated for 40 cycles, followed by a final elongation of 2 min at 72°C for 7 min. The amplicons were ligated into pCR 2.1-TOPO vector (Invitrogen, Carlsbad, CA. USA), purified, and then sequenced by using BigDye Terminator chemistry in an ABI Prim 310 genetic analyzer (Perkin-Elmer, Foster City, CA. USA).

### Phylogenetic Analysis

Genomic DNA sequences of the studied strains’ complete ITS and the partial gene sequences of the selected 4 exons (*CDC42, COXII, HSP90,* and *TUB*) were aligned with other *Lagenidium* and *Pythium* DNA sequences available at the National Center for Biotechnology Information by using ClustalW version 1.81 (http://www.clustal.org)with default settings and their alignments visually inspected. *Pythium* spp. groups a–c, e–g, and j (*15*) (Table) were used as outgroups. Phylogenetic and molecular evolutionary analyses were conducted by using MEGA6 (http://www.megasoftware.net). The aligned sequences were exported for parsimony analysis by using a heuristic search with tree bisection reconnection branch swapping (MEGA6) and distant analysis by neighbor-joining (MEGA6). We coded large insertions as 1 event by excluding all but 1 nt per insertion. The generated gaps were treated as missing data. Neighbor-joining analysis used either uncorrected distances or maximum-likelihood estimates of distance with a time-reversible model (6ST), and empirical base frequencies with no rate variation among sites, or a shape parameter of 0.5 γ-distribution with 4 rate categories. Branch support was estimated as percentage of parsimony tree (1,000 resampling, heuristic, branch swapping) or neighbor-joining trees (1,000 resampling, maximum-likelihood distances) within each branch. Concatenated DNA sequences of the 5 loci investigated were used in Bayesian analysis. We conducted the test in MrBayes version 3.2.1 ×64 (http://mrbayes.sourceforge.net) using the general time reversible + I + γ model, with 2 chains (1 heated), 2 runs, sampling every 100th generation for 1 × 10^6^ generations, and exclusion of the first 2.5 × 105 samples (the burn-in) before analysis. Support for branches was estimated as the percentage of parsimony tree (1,000 resampling, heuristic, nni branch swapping) or neighbor-joining tree (1,000 resampling, maximum-likelihood distances) containing the branch, as well as by determining the Bayesian probability estimated as the percentage of Bayesian trees possessing the branch after discarding the burn-in sample.

## Results

### Phylogenetic Analysis

**Figure 1:**
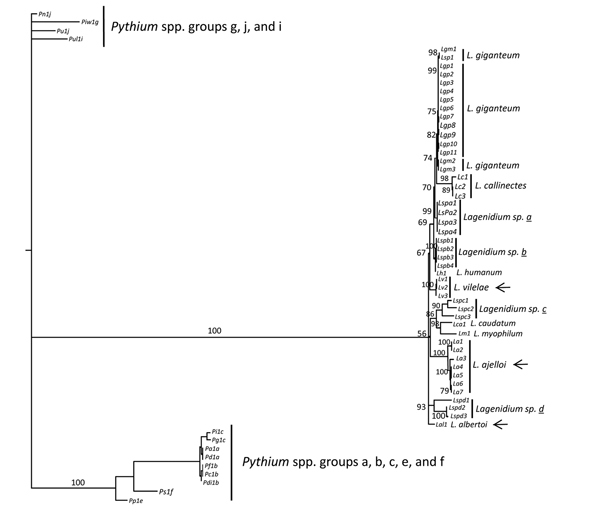
Bayesian phylogenetic analysis of concatenated 4 partial coding gene sequences (cell division cycle 42, cytochrome oxidase II, heat shock protein 90, and tubulin) and the complete internal transcribed spacers 1, 2, and 5.8S of *Lagenidium* DNA sequences. Thirteen *Pythium* species DNA sequences were included as the outgroup (groups a–c, e–g, j, and I [*16* ]; Table). Support on key branches is the Bayesian probability for that branch followed by the percentage of 1,000 bootstrap resampled datasets containing the branch in neighbor-joining analyses of maximum-likelihood distances followed by the percentage of 1,000 bootstrap resampled datasets containing the branch in parsimony analyses using heuristic searches. In this analysis, the DNA sequences of *L. giganteum* mosquito control (Lg 1–3) and a *Lagenidium* sp. recovered from a nematode in Taiwan (Ls1, Lsp1 = HQ395647) clustered with *L. giganteum* from mammals (Lg 1–10). The pathogen of crab *L. callinectes* (Lc) was the sister group to the cluster. Three *Lagenidium* mammalian pathogenic novel species (*L. ajelloi* = La, *L. albertoi* = Lal, and *L. vilelae* = Lv) were placed in 3 distinctive strongly supported clades (arrows). The accession numbers, the abbreviations used to identify each species, and the *Lagenidium* and *Pythium* spp. DNA sequences are shown in the Table. ATCC, American Type Culture Collection; CBS, Centraalbureau voor Schimmelcultures.

**Figure 2:**
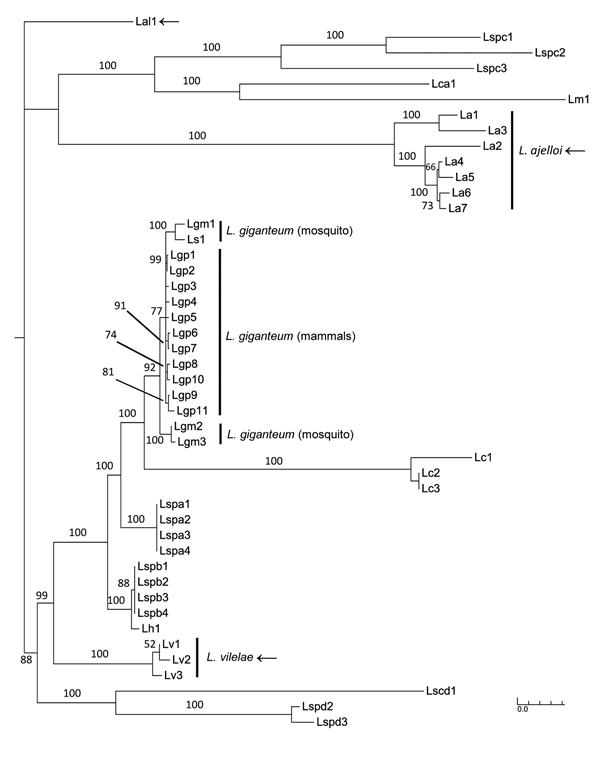
The Bayesian tree was constructed by concatenated aligned of *Lagenidium* spp. DNA sequences as in Figure 1 without outgroup to highlight the position of *L. giganteum* in the tree. *L. giganteum* from mammals (Lgp 1–11), *L. giganteum* mosquito control (Lgm 1–3 and Ls1 = HQ395647), and the novel species *L. ajelloi* = La, *L. albertoi* = Lal, and *L. vilelae* = Lv were placed in 4 strongly supported clades (arrows). Scale bar indicates nucleotide substitutions per site.

Using *Pythium* spp. groups a–c, e–g, and j (Table) as outgroups, we sorted the concatenated DNA sequences of 4 protein-coding genes and 1 ribosomal region in Bayesian and maximum-likelihood phylogenetic analyses 21 mammalian pathogenic *Legenidium* strains into 4 strongly supported clades in the combined analysis (Figure 1). In the largest of these clades, 11 of the 21 strains (Table) recovered from infected mammals form a monophyletic clade that includes other *L. giganteum* strains previously used to develop biological control agents of mosquitoes (Figure 1). Also included in this clade is an *L. giganteum* strain recovered from nematodes in Taiwan (Lsp1). The remaining 10 mammal-infecting *Lagenidium* strains were resolved with strong support into 3 independent clades that are basal to *L. giganteum* and identified in this study as *L. ajelloi*, *L. albertoi*, and *L. vilelae* (Figures 1, 2). These new species will be fully described elsewhere. Other clades of *Lagenidium* species, as yet unnamed, are also apparent in the phylogeny (Lspa to Ld; Figures 1, 2).

### Heat-tolerance and Mosquito Larva Infection Experiments

**Figure 3:**
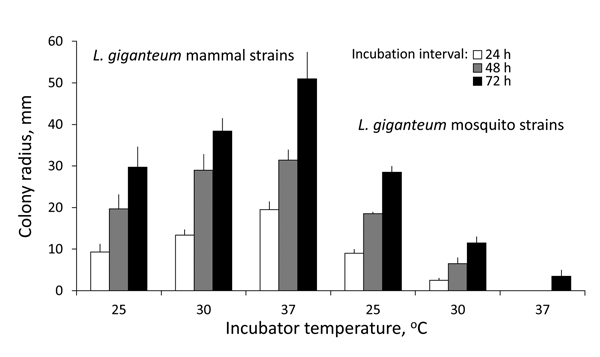
Cardinal temperatures of *Lagenidium giganteum* types in culture. Growth (mean colony radius and SEM, mm) of *L. giganteum* mammalian and mosquito strains at 3 temperatures at 24-, 48-, and 72-hour intervals postinoculation onto 2% Sabouraud dextrose agar. Repeated measures analysis of variance showed highly significant differences between strains (F1,33 = 165.0, p<0.0001) and a highly significant interaction of strain and incubation temperature across time intervals (F2,33 = 45.9, p<0.0001).

The 11 *L. giganteum* strains from mammalian hosts grew faster at 37°C and 30°C than at 25°C, whereas *L. giganteum* biological control strains (ATCC 36492 = ATCC 52675 and ATCC 48336) grew well at 25°C and poorly at 30°C and almost failed to develop at 37°C in growth experiments conducted in CMA and SDA media (Figure 3). The biological control strains barely developed at 37°C but survived, as shown by their subsequent growth, after being transferred to 25°C.

**Figure 4:**
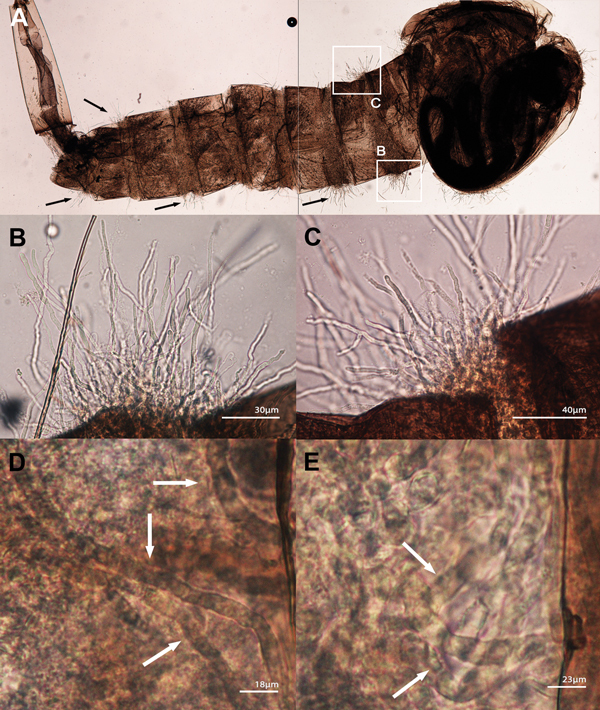
*Lagenidium giganteum* from mammal experimental infection using *Culex pipiens* mosquito larvae. A) Composite of 2 photographs showing an instar 3 *C. pipiens* larvae infected with 1 of the 5 tested strains of *L. giganteum* recovered from dogs with lagenidiosis (MTLA01, type strain). Note the mycelioid structures emerging from the infected larvae (arrows). B, C) Enlargements of the 2 white boxes in (A) showing details of the mycelioid structures emerging between the segments of the larvae. D, E) Aggressiveness of the invading mycelioid structures (arrows) within the body of *C. pipiens* larvae.

We tested the ability of the mammalian *L. giganteum* zoospores to infect mosquito larvae in 5 *L. giganteum* mammal strains (MTLA01, 03, 04, 05, and 10; Table) and 2 *L. giganteum* biocontrol strains (ATCC 48336 = Lgm2, ATCC 36492 = Lgm3; Table). The emerging mammalian pathogenic strains exhibited mosquito-infection capabilities similar to those of the strains approved by the US Environmental Protection Agency for mosquito control (Figure 4). At least 1 larvae per well was found infected. Infected larvae exhibited abnormal swimming behavior and died within 2 days (Figure 4). Of the 60 *C. pipiens* larvae (2 trials, 30 larvae per experiment), 12 were found infected at the end of both experiments (instar 3 or instar 4). Uninfected larvae continued their normal life cycle. Postmortem examination of infected larvae showed extensive infections with numerous hyphae spreading internally and emerging between segments (Figure 4, panels A–E). Under our experimental conditions, *L. giganteum* ATCC 36492 infected 18 of the 60 *C. pipens* larvae. In unexposed controls all larvae survived, larvae swam normally, and we found no evidence of infection. The identity of mammalian *L. giganteum* strains experimentally infecting mosquito larvae was confirmed by culture, DNA extraction, sequencing, and phylogenetic analysis at the conclusion of each experiment.

### Morphologic Characteristics of *L. giganteum* in Culture

**Figure 5:**
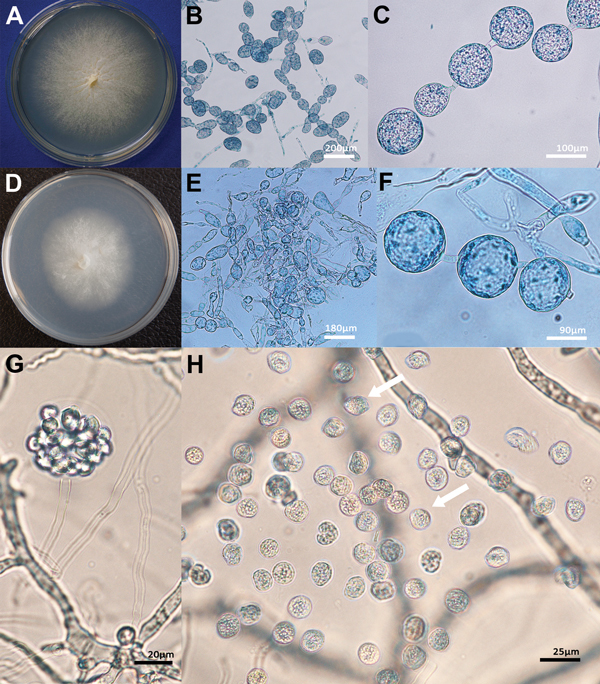
Morphologic features of isolates of *Lagenidium giganteum* mosquito control agent and *L. giganteum* mold from mammals. Panel A shows henotypic features in culture of the mammalian pathogen (ATCCMYA-4933, type strain) and panel D shows the biological control (ATCC 36492). The development of spherical and ovoid 40- to 170-μm swelling segments (panels B, C, E, F) was the main feature of both mammalian and biocontrol strains. Panel G shows A tubular body developed from an unseen segmented sporangium form a vesicle enclosing numerous zoospores in a mammalian *L. giganteum* strain. The kidney-shaped zoospores before release (G) and after release (H, arrows) agree with those in the original description of *L. giganteum* by Couch (*10*). ATCC, American Type Culture Collection.

Submerged colonies of both *L. giganteum* types (biological control and mammalian strains) are colorless or yellow with few aerial mycelia that present irregular radiating and undulating patterns on any of 3 media (CMA, BHI, or 2% SDA) (Figure 5, panels A, D). Typically, the elongated mycelium comprises ovoides or spherical 30- to 40-μm diameter segments forming chains and connected by broad to slender isthmuses; hyphae also form long irregular segments of >400 μm (Figure 5, panels B, C, E, F). We did not find oogonia in the evaluated strains. In liquid sporulation medium, hyphal segments develop 1–2 discharge tubes that produce terminal vesicles containing reniform zoospores 9–10 μm wide × 10–12 μm long (Figure 5, panel G). Zoospores were released in both mammal and mosquito *L. giganteum* strains after the discharge vesicle was disrupted (Figure 5, panel H), which then swam for 10–20 minutes before encysting. Germ tubes developed from encysted zoospores within 10–20 minutes after encystment.

## Discussion

We detected a novel group of mammalian pathogenic *L. giganteum* strains unique for their capacity to experimentally infect invertebrate hosts and cause life-threatening infections in healthy lower animals and humans (*5*,*7*). Placement of both heat-sensitive (affecting mosquito larvae) and heat-tolerant (affecting mammals) *L. giganteum* strains in a strongly supported monophyletic clade in Bayesian analysis corroborates the taxonomic and phenotypic attributes shared by both types in this and other studies (*3**–**5**,**10**–**15*). In addition to *L. giganteum*, 3 other clades harbor mammal isolates: *L. ajelloi*, *L. albertoi*, and *L. vilelae* (Figures 1, 2). Strains in the remaining clades are from invertebrates, whether from nematodes (the 4 unnamed species, *L. spa*, *L. spb*, *L. spc*, and *L. spd* [Table]), crab eggs (*L. callinectes*), or shrimp (*L. myophilum*) (*17*). *Lagenidium* strains in the 3 clades closest to *L. giganteum* infect invertebrates, as can all isolates of *L. giganteum*, suggesting that the mammal-infecting *L. giganteum* isolates recently evolved the ability to infect warm-blooded hosts. However, the capacity to infect mammals is not unique to *L. giganteum*, and knowing that each of the 3 other mammal-infecting *Lagenidium* clades has closely related relatives that infect invertebrates, it seems likely that the trait of infecting mammals has arisen several times, independently, in the genus *Lagenidium*. From its name, 1 species, *L. humanum*, might be expected to be among the mammal parasites, but it has been isolated only from the dead skin of humans or snakes and cannot grow at 37°C (*9*). Regarding the trait of heat tolerance, species that are isolated from mammals, *L. ajelloi*, *L. albertoi*, *L. giganteum*, and *L. vilelae* also can grow at 37°C, but judging from cultures recovered from lower animals, some strains associated with invertebrates also might lack the heat-tolerance trait. *Lagenidium* strain Centraalbureau voor Schimmelcultures (CBS) 127042 recovered from nematodes and phylogenetically linked to *L. vilelae* may be a good example of this unusual feature (Figure 1).

Emergence of mammalian pathogens often is accompanied by host switching in a zoonotic context from 1 vertebrate species to another (*18*,*19*), but range extension from invertebrates to vertebrates as hypothesized here is rare. Waterfield et al. (*20*) outlined 3 scenarios in which bacteria pathogenic to humans might have evolved from pathogens associated with invertebrates, but the time for the process was thousands of years, whereas in our second scenario, we describe a process that might have happened within a decade. The 2 phenotypes of heat-tolerance and mammal pathogenicity appear to have repeatedly evolved in the genus *Lagenidium*, and several recent studies have shown that members of Oomycota can acquire pathogenicity genes by horizontal gene transfer (*21*–*23*). Before their release as agents of biocontrol, *L. giganteum* (ATCC 52675 = ATCC 36492) (*12*) and other strains proposed as biocontrol agents of insect were shown not to infect other arthropods and mammals (*14*,*15*). However, experimental infection of mammals by the other Oomycota pathogen, *P. insidosum*, is difficult to achieve, e.g., only rabbits have been successfully, experimentally infected by this pathogen of cats, cattle, dogs, horses, and humans (*2*). This experience indicates that the safety of *Lagenidium* species as pathogens of hosts other than mosquito might be difficult to assess because of a general difficulty over experimental infection of mammals by Oomycota species.

The evolutionary relationships among *L. giganteum* strains should be more thoroughly examined by using population genomics, which could lead to discovery of the basis of adaptation to specific hosts, as has been shown for other adaptive phenotypes in other filamentous microbial eukaryotes (*24*). The resent transcriptome analysis of a strain of *L. giganteum* recovered in nature from mosquito larvae further strengthens the relevance of our findings (*25*). The use of varieties or subspecies to name these 2 populations of *L. giganteum* was avoided until more comprehensive genomic data shed light on their true evolutionary relationships. Future genomic studies also could discriminate between the scenarios of gaining novel traits in specific ecologic niches or revealing types with hidden capabilities, both of which are of environmental and public health concern when the traits include putative pathogenicity of humans.
